# Exploring reasons for non-vaccination against human papillomavirus in Italy

**DOI:** 10.1186/s12879-014-0545-9

**Published:** 2014-11-11

**Authors:** Cristina Giambi, Fortunato D'Ancona, Martina Del Manso, Barbara De Mei, Ilaria Giovannelli, Chiara Cattaneo, Valentina Possenti, Silvia Declich, Local Representatives for VALORE

**Affiliations:** Communicable Disease Epidemiology Unit; National Centre for Epidemiology, Surveillance and Health Promotion, Istituto Superiore di Sanità, Viale Regina Elena 299, 00161 Rome, Italy; European Programme for Intervention Epidemiology Training (EPIET), European Centre for Disease Prevention and Control, Stockholm, Sweden; Unit of Training and Communication; National Centre for Epidemiology, Surveillance and Health Promotion, Istituto Superiore di Sanità, Viale Regina Elena 299, 00161 Rome, Italy

**Keywords:** Human papillomavirus infection, Vaccines and immunisation, Uterine cervical cancer, Immunisation programs, Reasons for non-vaccination, Acceptance

## Abstract

**Background:**

In Italy, free-of-charge HPV vaccination is offered to 11-year-old girls since 2007. The National Immunization Plan established the target coverage at a minimum of 70%; it should increase to 95% within 3-year time frame. In 2012, four year after the introduction of HPV vaccination, coverage was stable at 69%. We conducted a national cross-sectional study to explore barriers to vaccination in Italy.

**Methods:**

Vaccination services selected, through the immunization registries, a sample of unvaccinated girls born in 1997 or 1998 and posted to their families a 23-items questionnaire inquiring barriers to vaccination, HPV knowledge, source of information on HPV, perception of risk of contracting HPV, advice from consulted health professionals on HPV vaccination.

**Results:**

We analysed 1,738 questionnaires. Main barriers were fear of adverse events (reported by 80% of families), lack of trust in a new vaccine (76%), discordant information received by health professionals (65%) and scarce information on HPV vaccination (54%). Overall, 54% of families replied correctly to more than half of 10 questions exploring knowledge on HPV vaccination. Families with a high knowledge score were more likely to live in Northern and Central Italy, be Italian, have a high educational level, include a mother who attended cervical screening regularly and consult more information sources. Although paediatricians/general practitioners and gynaecologists were considered the most trusted source of information by 79% and 61% of respondents, they were consulted only by 49% and 31%. Among parents who discussed vaccination with a physician, 28% received discordant advices and 31% received the recommendation of accepting vaccination.

**Conclusions:**

Fear of adverse events, discordance of information and advices from physicians, and scarce information were the more commonly reported barriers to HPV vaccination. Health professionals played a key role as information providers, thus they must be better trained to provide clear notions. Training needs to include the development of communication skills; transparent discussion about the pros and cons of vaccination may reduce fear of adverse events and increase trust in vaccination. The creation of a public health network around vaccination would allow sharing information and attitudes on vaccinations, so that homogeneous messages could reach the target population.

**Electronic supplementary material:**

The online version of this article (doi:10.1186/s12879-014-0545-9) contains supplementary material, which is available to authorized users.

## Background

Human Papillomavirus (HPV) vaccination represents an important opportunity for primary prevention of cervical cancer (CC). HPV vaccines have a high efficacy against cervical pre-cancer lesions if given to females before they are exposed to the virus [1], therefore the World Health Organization (WHO) recommends to offer HPV vaccination to pre-teen girls.

As of December 2012, 22 of 31 EU/EEA countries had implemented HPV vaccination. Target age, financing and vaccine delivery differ among countries [2]. In 2012, European countries report coverage varying from 17% to 84% [3].

In Italy, since 2007, public immunization services of all Local Health Units (LHUs) actively invite 11-year-old girls for free-of-charge HPV vaccination [4]; most LHUs plan one or more reminders for non-respondents. In spring 2007 the Italian Ministry of Health started a nationwide information campaign; most regions also conducted additional campaigns. The 2012-2014 National Immunization Plan established the target coverage rate at a minimum of 70% of 11 year-old girls vaccinated with three doses of HPV vaccine; it should increase to 95% within 3-year time frame [5]. On 30/06/2013, 4 years after the introduction of HPV vaccination, the national immunization coverage of the first cohorts called for vaccination (1997-1999) was stable at 69% [6]. Adolescents represent a difficult target for vaccination; an Italian survey carried out in 2008 found that only 53% of 15-year-old adolescents had received the diphtheria-tetanus booster dose planned at 11-15 years [7].

Many studies explored factors influencing HPV vaccination uptake and parental attitudes towards HPV vaccination. Three 2012-13 reviews [8]-[10] indicated that (a) recommendation from a doctor is the main driver of vaccination, (b) safety is a key parental concern, (c) HPV vaccine-related knowledge is positively associated with vaccination uptake and (d) school-based immunization programs increase vaccination coverage.

In Italy no research on barriers to HPV vaccination targeting parents of unvaccinated girls was available at national level. We conducted a survey among a sample of families of unvaccinated girls to explore reasons for non-vaccination. Understanding parental reasons for not vaccinating their daughters can help public health authorities to implement interventions increasing HPV vaccine acceptance.

## Methods

### Study design

We conducted a cross-sectional study in the period November 2011-July 2012 as part of a national project (VALORE), coordinated by the National Institute of Public Health (Istituto Superiore di Sanità, ISS) and funded by the Ministry of Health.

The survey addressed families of unvaccinated girls born in 1997 or 1998 who were offered vaccination in 2008-2010. We invited all 143 LHUs to participate (distributed in 21 regions/autonomous provinces). We excluded LHUs that were implementing an HPV vaccine catch-up programme targeting birth cohorts 1997 or 1998 at the time of the study.

LHUs identified unvaccinated girls through the immunization registries. For calculating the sample size we assumed a precision of 10%, an alpha error of 5% and, given that current reference literature data was not available, a prevalence of 50%. According to these parameters, we needed a sample of at least 96 units for each LHU/region to provide sufficient power to generate estimates at local/regional level. Assuming a response of 30%, we randomly selected 320 girls from each list of unvaccinated girls. If unvaccinated girls were fewer than 320, we invited all of them.

### The questionnaire

We developed a 23-item questionnaire (mainly close-ended, 15 minutes needed for administration) inquiring about: 1) demographic information, 2) vaccination status, 3) barriers/reasons for non-vaccination, 4) HPV knowledge, 5) source of information on HPV, 6) perception of risk that their daughter could contract HPV, 7) intention to have their daughter vaccinated in the future, 8) advice from consulted health care workers (HCWs) on HPV vaccination, 9) parents' socio-demographic and behavioural characteristics. Copies of the questionnaire (in Italian) are available from the authors.

To investigate barriers to vaccination, we proposed a list of 23 possible reasons for non-vaccination and asked participants to indicate how much each factor had influenced their decision of non-vaccination on a 4-point scale (A lot/sufficiently/a little/not at all). To explore HPV knowledge, we proposed ten statements on HPV infection/vaccination, with three options available (True/False/Do not know). Regarding sources of information, we proposed a list and asked participants who provided them information (they could indicate more than one source) and which sources they considered more trusted (they could indicate a maximum of three sources). For the other questions, answers with multiple options were proposed and parents could indicate one.

We administered the questionnaire to a convenience sample of parents to ensure clarity and ease of administration, collected their comments and modified accordingly.

### Data collection

In the period January-March 2012, vaccination services posted to the selected families a letter explaining the purpose of the study, the self-administered questionnaire, a stamp-addressed reply envelope to return the questionnaire to ISS and the invitation to the immunization service to get free-of-charge HPV vaccination. Since the survey was voluntary and anonymous, completing the questionnaire was considered as consent to participate. The national ethics committee of the ISS approved the study protocol.

### Statistical analysis

We summarized categorical variables using frequencies and proportions and used Chi-square test, Fisher's exact test and Chi-square for trend to compare proportions. We defined statistical significance as a 2-tailed *p*-value of <0.05. For the purpose of the uni-variate and multivariable analysis, we dichotomized the following variables: educational level (at least one parent with high school or college degree/other), age (at least one parent <45 years/other), occupation (at least one employed parent/other), mother's attitude toward Pap test (regularly undergone/done once or never) and other accepted paediatric vaccinations (all proposed vaccinations/only some vaccinations or none). We computed a dichotomized knowledge score based on the number of correct answers on HPV infection and vaccination (≤5/>5) (outcome). We calculated odds ratios and their 95% confidence intervals (95% CI) to assess the association between selected variables and the outcome (knowledge score). We included into a logistic regression model the variables showing potentially interesting associations with the outcome and we used a forward stepwise procedure with the method of the likelihood ratio test for goodness-of-fit in order to control for possible confounding factors. At each step, a p-value of 0.10 was used as entry criterion and a p value of 0.15 as removal criterion. We used the statistical package STATA 11.2 to analyse data (Stata Corporation, College Station, Texas, USA).

## Results

### Study participation

Fifty-six LHUs in ten regions participated in the survey: 32 in the North, 16 in the Centre and 8 in the South. Vaccination services posted 14,099 letters and 2,110 questionnaires returned to ISS, with a response of 15% (16.5% in the North, 12.2% in the Centre and 10.3% in the South). We excluded 372 questionnaires: 25 were filled incorrectly, 57 were sent to wrong cohorts and 290 to girls already vaccinated against HPV. Overall, we analysed 1,738 questionnaires.

The number of letters returned to the sender because of unknown recipient was available for 39/56 LHUs; 7.3% of letters (816/11,187) returned to the vaccination centre, with a wide range among LHUs (0-22%).

### Study population

Ninety-nine percent of girls were Italian; 80% lived in the North of Italy; 99% had received other paediatric vaccinations. In most cases (74%) the mother filled the questionnaire; 81% of girls had at least one parent with secondary school or university degree; 88% of mothers underwent Pap-test regularly (Table 1). Before the invitation to this project, 7% of families had not received any call for HPV vaccination from immunization services.

**Table 1:** **Characteristics of the study population**; **Italy**, **2012**

		n	%
Birth Cohort (N = 1738)	1997	167	9.6
	1998	1571	90.4
Geographic area (N = 1729)	North	1390	80.4
	Centre	207	12.0
	South	132	7.6
Nationality (N = 1726)	Italian	1659	96.1
	Foreign	67	3.9
Other paediatric vaccinations (N = 1715)	Yes, all proposed by paediatrician/vaccination service	1393	81.2
	Yes, some of them	304	17.7
	No	18	1.04
MMR vaccination (N = 1692)	1 dose	332	19.6
	2 doses	1124	66.4
	No MPR vaccination	191	11.3
	I do not remember	45	2.7
Parents' nationality (N = 1705)	Both Italian	1516	88.9
	Both foreign	62	3.6
	Italian/Foreign	30	1,8
	Other	97	5.7
Parents' age (N = 1712)	< 45 years, both parents	498	29.1
	< 45 years, both parents	727	42.5
	< 45/ ≥45	397	23.2
	Other	90	5.3
Parents' education level (N = 1717)	High school or college degree, both parents	879	51.2
	High school or college/primary or middle school degree	452	26.3
	Primary or middle school degree, both parents	290	16.9
	Other	96	5.6
Parents' status of employment (N = 1663)	Both parents employed	1111	66.8
	Only one parent employed	499	30.0
	Both parents unemployed	17	1,0
	Other	36	2.2
Adolescents (>16 years) in the family (N = 1714)	≥1	642	37.5
	0	1072	62.5
Parents' smoking habit (N = 1727)	At least 1 non/ex smoker parent	1614	93.5
	Other	113	6.5
Mother' attitude toward Pap test (N = 1673)	She regularly undergoes	1469	87.8
	She underwent at least once	160	9.5
	Never done	44	2.7
Parent who filled the questionnaire (N = 1713)	Mother	1265	73.8
	Father	76	4.4
	Both parents	372	21.7

### Barriers to HPV vaccination

**Figure 1:**
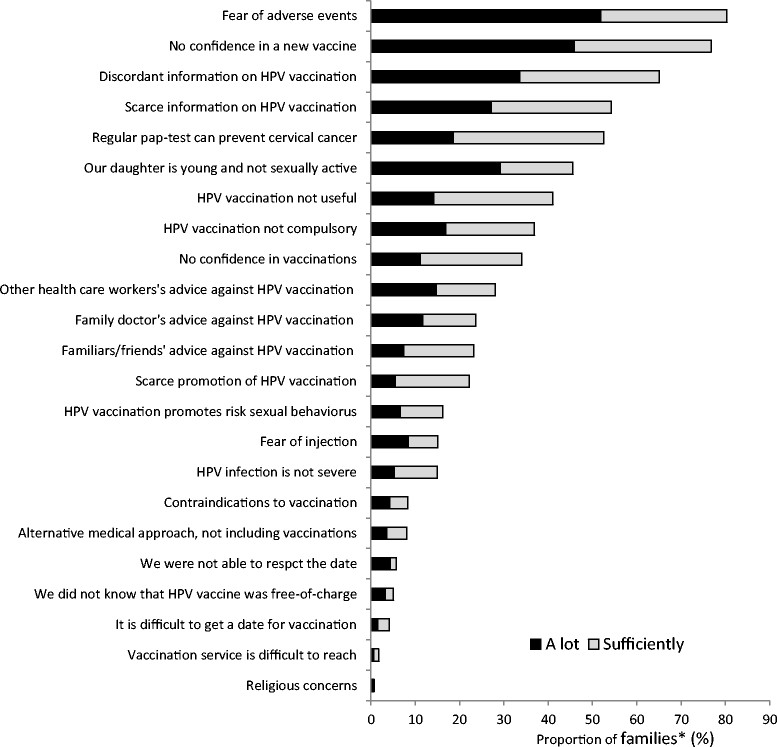
**Barriers to HPV vaccination in a sample of unvaccinated girls; Italy, 2012.** Notes for figure 1: * Proportion of families declaring that each potential barrier to HPV vaccination had influenced “a lot/sufficiently” the decision not to accept their daughter's vaccination.

The main reasons that influenced a lot/sufficiently the decision not to accept HPV vaccination were fear of adverse events (reported by 80% of families, 95% CI 78.4-82.3), lack of trust in a new vaccine (76%, 95% CI 74.7-78.8), discordance of information received by HCWs on HPV vaccination (65%, 95% CI 62.6-67.4) and scarce information on HPV vaccination (54%, 95% CI 51.8-56.7) (Figure 1). These four factors represented the main barriers to vaccination, also when stratifying by area and educational level.

A negative advice concerning HPV vaccination from the family doctor or other health professionals was indicated by 24% (95% CI 21.5-23.6) and 28% (95% CI 25.8-28.5) of parents; combining these two questions, 39% (95% CI 36.9-41.7) of families reported that a negative advice from a physician influenced the decision of non-vaccination. The belief that the age for vaccination was too young was reported by 45% (95% CI 43.1-48.1). Only 16% (95% CI 14.4-18.1) feared that vaccination would encourage more risky sexual activity. Issues linked to the access to vaccination services influenced marginally the acceptance (Figure 1).

Out of 1,708 families, 827 (49%) confirmed the intention to refuse HPV vaccination for their daughter in the future, 504 (29%) had not taken a decision yet and 377 (22%) intended to accept vaccination.

### Knowledge on HPV infection and vaccination

Most respondents were aware of the causal relationship between HPV and CC (93%), the high frequency of HPV infections (70%), the sexual transmission of HPV (77%) and its asymptomatic nature (72%). Few respondents were aware of the transient nature of most HPV infections (19%), the possibility for men to be infected (37%) and the connection between HPV and genital warts (29%) (Table 2). Each family replied correctly to a mean of 5.6/10 questions. Compared with others, families with a high knowledge score were more likely to live in Northern and Central Italy, be Italian, have a high educational level, include a mother who attended cervical screening regularly and consult more information sources (Table 3).

**Table 2:** **Parents**' **knowledge on HPV infection and vaccination**; **Italy**, **2012**

Questions exploring knowledge	n/N	%*
HPV may cause cervical cancer (T)	1571/1690	93.0
HPV is a sexually transmitted disease (T)	1295/1675	77.3
HPV may infect you without symptoms (T)	1195/1669	71.6
HPV infections are rare (F)	1167/1661	70.3
HPV vaccines work well if given before sexual debut (T)	1045/1655	63.1
HPV vaccines protect against all HPV types (F)	981/1670	58.7
Sometimes HPV infections can last years (T)	899/1646	54.6
Only women can be infected by HPV (F)	615/1647	37.3
Genital warts are caused by HPV (T)	471/1633	28.8
Most HPV infections resolve spontaneously (T)	320/1662	19.3

*Proportion of parents that replied to each question correctly. The correct answer is indicated among bracket (T = true, F = false).

**Table 3:** **Determinants of high score of knowledge on HPV infection and vaccination**; **Italy**, **2012**

Variables			Univariate analysis		Logistic regression	
		n (%)	OR_crude_	95%CI	OR_adj_	95%CI
Geografical area	North	747 (55.0)	2.06	1.42 - 2.99	1.79	1.17 - 2.75
	Centre	115 (56.7)	2.20	1.40 - 3.47	1.73	1.04 - 2.89
	South	48 (37.2)	1	-	1	-
Nationality	Italian	901 (55.4)	4.68	2.52 - 8.69	3.03	1.55 - 5.91
	Foreign	13 (21.0)	1	-	1	-
Other paediatric vaccinations	All proposed vaccinations	724 (53.0)				
	Only some vaccinations /None	186 (58.1)				
Adolescents (>16 years) in the family	≥1	868 (54.8)				
	0	43 (39.1)				
Parents' education level	At least 1 parent with high school or college degree	821 (59.7)	3.76	2.87 - 4.92	3.10	2.33 - 4.15
	Other	88 (28.3)	1	-	1	-
Parents' age	at least 1 parent <45 years	464 (50.5)	1.34	1.10 - 1.62		
	Other	440 (57.7)	1	-		
Parents' status of employment	At least 1 parent employed	863 (54.5)				
	Other	22 (45.8)				
Parents' smoking habit	At least 1 non/ex smoker parent	808 (55.2)	1.89	1.27 - 2.81		
	Other	97 (46.0)	1	-		
Mother' attitude toward Pap test	Regularly undergone	849 (58.7)	3.99	2.86 - 5.57	2.66	1.85 - 3.84
	Done once or never	52 (26.3)	1	-	1	
Number of source of information	0-1	156 (33.0)	1	-	1	-
	2-3	525 (58.3)	2.84	2.25 - 3.59	2.30	1.79 - 2.96
	≥ 4	229 (72.9)	5.47	4.00 - 7.49	3.96	2.84 - 5.53

Determinants of high score of knowledge * on HPV infection and vaccination; Italy, 2012.

*We computed a dichotomized knowledge score based on the number of correct answers on HPV infection and vaccination: “high” if > 5 correct answers; “low” if > 5 correct answers

Out of 1664 families, 561 (34%) considered their daughter at risk of contracting HPV, 226 (14%) did not perceive any risk and 877 (53%) were not able to reply. The proportion of families perceiving the risk increased from 9.1% among those families that gave one correct answer to 59.2% among those who replied to all questions correctly (p < 0.0001, Chi-square for trend).

### Sources of information on HPV infection and vaccination

Ninety-three parents (5%) had never heard about HPV. Paediatrician/general practitioner represented the most commonly consulted source of information on HPV vaccination; the most trusted sources of information included paediatrician/general practitioner and gynaecologist. Although 79% and 61% of families trusted more their paediatrician/general practitioner and their gynaecologist, only 49% and 31% of the respondents had consulted these specialists (Table 4).

**Table 4:** **Sources of information on HPV infection and vaccination**; **Italy**, **2012**

		n	%
Used sources of information (N = 1616^)	Paediatrician/general practitioner	794	49.1
	Friends/family members	651	40.3
	Internet	553	34.2
	Gynaecologist	495	30.6
	Newspaper/leaflet/poster	434	26.9
	Vaccination services	403	24.9
	Radio/television	326	20.2
	School	150	9.3
	Mother and child health centers	93	5.8
	Pharmacist	39	2.4
	Other	109	6.7
Mutually exclusive sources (N = 1565)	Health care workers plus others	870	55.6
	Health care workers only*	337	21.5
	Other sources only**	358	22.9
Number of sources of information (N = 1565)	0-1	486	28.4
	2-3	906	53.0
	≥4	317	18.6
Most trusted sources of information (N = 1640)	Paediatrician/general practitioner	1288	78.5
	Gynaecologist	1004	61.2
	Vaccination services	564	34.4
	Internet	342	20.9
	Mother and child health centers	245	14.9
	Friends/family members	67	4.1
	Pharmacist	36	2.2
	Other	142	8.7

^Parents that had never heard about HPV were excluded from the denominator

*gynaecologist, paediatrician/general practiotioner, vaccination service, mother and child health centres, pharmacist

**friends/family members, Internet, newspaper/leaflet/poster radio/television, school

Overall, 870 (56%) families consulted health professionals and other sources of information, while 358 (23%) had not received information by any physician; 906 (53%) indicated learning on HPV vaccination from 2-3 sources (Table 4).

Of 1,710 families, 600 (35%) felt sufficiently informed regarding HPV infection and vaccination; 787 (46%) did not feel enough informed and 323 (19%) were not able to give an opinion. Of 1,562 families, the information received by health professionals were considered appropriate to decide on HPV vaccination by 622 (40%); scarce and generic by 314 (20%); unclear regarding the vaccine mechanism by 278 (18%) and unclear on safety issues by 623 (40%).

### Health professionals' advice regarding HPV vaccination

**Figure 2:**
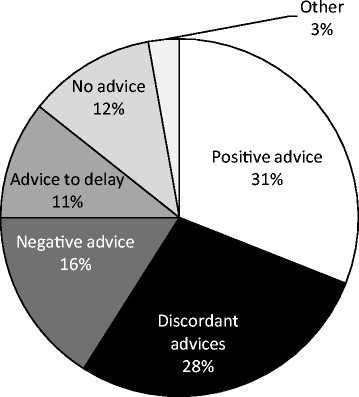
**Advices concerning HPV vaccination received by consulted health professionals; Italy, 2012.**

Out of 1,703, 345 (20%) parents did not discuss about HPV vaccination with any health professional. Among the 1,358 parents who addressed the issue of HPV vaccination with a physician, 377 (28%) received discordant advices; only 421 (31%) parents received the recommendation of accepting HPV vaccination (Figure 2).

## Discussion

In our study, participants: 1) feared the occurrence of adverse events of HPV vaccination, 2) were not well informed and aware of HPV infection and vaccination, 3) had received discordant advices and information on HPV vaccination from health professionals.

Regarding the first point, concern about side effects was the main barrier to acceptance, in accordance with previous surveys [10]-[12]. The incidence of many vaccine-preventable diseases has decreased, thus the risk of the potential vaccine adverse effects is perceived to be greater than the risk of the disease. The provision of complete information on vaccine safety by well-trained HCWs could encourage acceptance. Pre-vaccination counselling has to cover not only the benefits of vaccination and the risks related to the disease, but also consider doubts, fears, and risk of severe side-effects. To increase trust in vaccination, information needs to be clear, detailed and evidence-based. Communicating that vaccination entails no risk may, paradoxically, lead patients to view vaccines as more risky [13].

Concerning the second point, the level of knowledge on HPV infection/vaccination was low. Since responders had received an invitation for their daughter's vaccination, they could have been more likely to seek HPV-related information. However, only 54% of families replied correctly to more than half questions. Our data suggest that many respondents do not understand the link between HPV and genital warts, the possibility for men to be infected and the transient nature of most HPV infections; other surveys conducted in Italy on the same topic [14]-[16] confirmed that knowledge on these aspects is not consolidated.

Only one third of families felt enough informed and the same proportion perceived the risk that their daughter could acquire the infection. Although knowledge does not predict behaviour's modification, it is a prerequisite for an aware decisional process on vaccination. A higher vaccine-related knowledge was positively associated with HPV vaccination uptake [8],[9] or intention of undergoing HPV vaccination [15]. According to Trim et al. [10], concern about the potential risk of cancer and believing their daughters might contract HPV and related diseases drive HPV vaccination. Also Donati et al. [15] found that women who were fairly and very worried about getting an HPV infection had a higher probability of accepting vaccination.

Regarding the third point, discordant information and advice received by different health professionals hindered vaccination for 65% of families. Only 31% received positive advice from a physician. Studies reported that a proportion of clinicians had substantial concerns about promoting vaccination, particularly for younger age groups [17]. National [15],[18] and international [9]-[11],[19],[20] literature reports that health care provider recommendation is one of the main factors driving acceptance. To guide families towards an informed decision regarding vaccination, physicians need to provide complex explanations (e.g., why the vaccine is offered only to females, why it has to be administered to girls before sexual debut and the fact that the currently available vaccines only prevent certain HPV oncogenic types). Moreover HPV is a sexually transmitted infection and the promotion of programs regarding sexual issues can be more difficult than other public health actions, above all if targeting pre-adolescent girls, mostly not sexually active.

In our study, family doctors and gynaecologists were considered the most trusted sources of information. However, there was a discrepancy between consulted and preferred sources of information on HPV vaccination: 30% of families that considered them as the most trusted source of information did not receive information on HPV from these professionals. A similar discrepancy emerged from a knowledge-attitude-practice survey among young adult Italian women [15]. Since parents preferred paediatricians, general practitioners and gynaecologists as sources of information, an active offer of information from these specialists at every contact with parents and girls could guide a conscious decision on HPV vaccination.

Only 25% of families got information on HPV vaccination from immunization services providers and 34% mentioned them among the most trusted sources of information. Another Italian survey reported that only 12% of young adult women would have liked to receive information on HPV vaccination by physicians working in vaccination service [15]. Even though vaccinations are offered and administered in public vaccination clinics in Italy, population do not consider immunization services providers as a reference point for vaccination.

HCWs represented the most used source to get information on HPV, but 56% of parents actively searched other sources and 23% of families did not consult any health professional and obtained information from alternative sources only. Overall, 34% of families used the Internet to obtain information on HPV. The vaccine criticism movement took advantage of the Internet's ability to reach parents [21]. A 2012 systematic review of Italian Internet pages on vaccination risks [22] reported that 67% of 144 pages analysed, often on top positions, was against vaccinations and 24% of them were written by physicians. All institutional pages (12%) were pro-vaccinations but they did not score high in visibility. Therefore, a more active presence of health institutions on Internet could “contrast” positions against vaccination, providing reliable and clear information to health professionals and population.

Caution should be exercised when comparing our results to other studies exploring knowledge on HPV vaccination because of different methodologies and target population. Contrarily to ours, a survey on this topic conducted by *Censis*[14], an Italian social study and research institute, that involved 3500 women aged 18-55 years, found that mass media are the largest source of information on HPV vaccination. The same finding came out from another national survey carried out on a sample of 667 women aged 18-26 years [15]. A possible explanation of the discordance with our results is that our sample is represented by families that have received an invitation for their daughters to be immunised against HPV and, therefore, they could have been more likely to consult health professionals to get a decision. A survey conducted in Italy among 987 young women supports this finding [23]; the authors found that magazines/books and television represent the main source of information for the over-18's; instead the under-18's are mostly informed by HCWs (general practitioners and paediatricians), probably because the under-18's are guided by parents and are therefore accompanied to healthcare services.

Finally, we realized that invitation for HPV vaccination from LHUs did not reach some adolescents. In fact, 7% of families had not received the active call for HPV vaccination and 7% of our letters returned to the sender. This suggests that data from the immunization archives could be outdated. The improvement of computerized immunization registries linked to resident/health lists, as addressed in the National immunization Plan [5], represents a priority that will improve the active call of immunization target population and coverage assessment.

The limitations of our study include: scarce geographic representativeness of the sample (most families lived in Northern Italy), lack of a comparative group of parents of vaccinated girls and low response (15%). The low response might have introduced selection bias; respondents may not represent all unvaccinated girls' families (socio-demographic information of non-respondents are unknown). Because of the low response, the minimum sample size was achieved only in few LHUs; as a result, data could only be analysed at the national level. Previous experiences suggested that posting a self-administered questionnaire could lead to a lower response than face-to-face interviews. However, self-administration represented the easiest procedure to propose participation at the national level without overloading vaccination services.

## Conclusions

Fear of adverse events, discordant information received by HCWs, and scarce information were the more commonly reported barriers to HPV vaccination. It suggests that, in our study, the decision of non-vaccination might come from lack or discordant information on HPV vaccination rather than from a conscious intention to decline vaccination. As HCWs played such a key role as information providers, they must be better trained to provide clear and homogeneous information to adolescents and their parents. Such training should include the development of communication skills; openness and transparent discussion about the pros and cons of HPV vaccination as well the use of appropriate communication strategies may help reducing fear of adverse events and increasing trust in vaccination among parents. Efforts are also needed to stimulate collaboration among health professionals; the creation of a public health network around vaccination would allow sharing information and attitudes on vaccinations, so that homogeneous messages could reach the target population. Such network would enforce the role of public vaccination clinics as reference point for HCWs and general population and encourage an active role of general practitioners, paediatricians and gynaecologists for vaccination promotion. Finally, offering to adolescents a “package of vaccinations” (including HPV, diphtheria-tetanus-pertussis, meningococcal, rubella, measles and varicella vaccination, if susceptible) and using all access points in the health system to verify their coverage and offer lacking vaccines could represent an integrated method to increase acceptance among adolescents.

## Authors' information

Multiple affiliations of local representatives for VALORE listed at: http://www.epicentro.iss.it/problemi/hpv/pdf/Allegato3_Gruppo%20Lavoro%20VALORE.pdf.

## Authors’ original submitted files for images

Below are the links to the authors’ original submitted files for images. Authors’ original file for figure 1 Authors’ original file for figure 2
