# Supplementary figures and images for: Exploring reasons for non-vaccination against human papillomavirus in Italy
Source: BMC Infect Dis. 2014 Nov 11;14:545. doi: 10.1186/s12879-014-0545-9 (PMC4233085; doi:10.1186/s12879-014-0545-9)

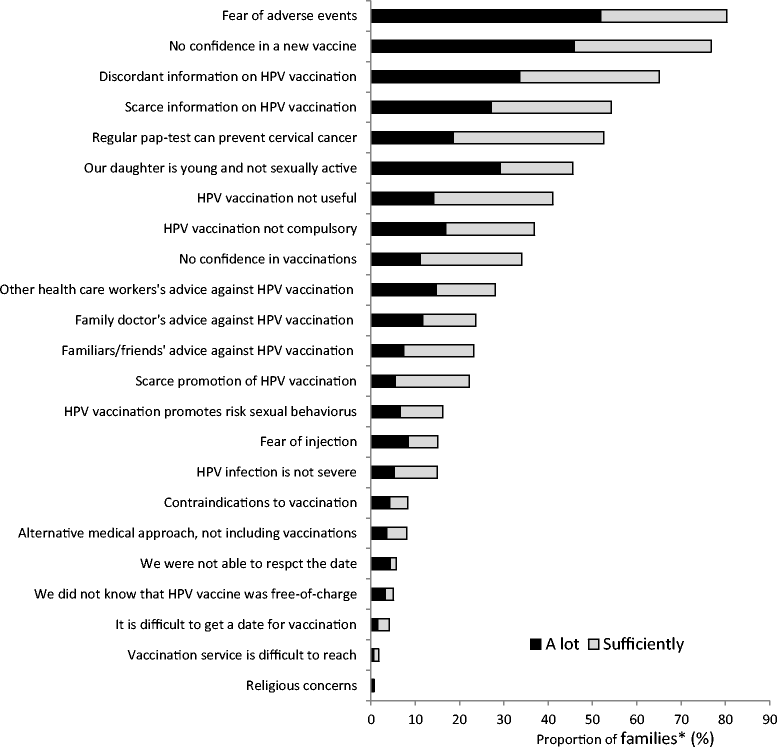

Supplement: Supplementary file 1 — Authors’ original file for figure 1 [file 12879_2014_545_MOESM1_ESM.gif]

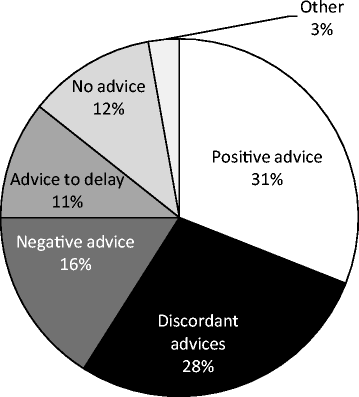

Supplement: Supplementary file 2 — Authors’ original file for figure 2 [file 12879_2014_545_MOESM2_ESM.gif]
